# Choice, control, and animal welfare: definitions and essential inquiries to advance animal welfare science

**DOI:** 10.3389/fvets.2023.1250251

**Published:** 2023-08-02

**Authors:** Maisy D. Englund, Katherine A. Cronin

**Affiliations:** ^1^Animal Welfare Science Program, Lincoln Park Zoo, Chicago, IL, United States; ^2^Department of Psychology, Georgia State University, Atlanta, GA, United States

**Keywords:** choice, control, animal welfare, agency, psychological wellbeing, affective states

## Abstract

The ways in which humans can support good welfare for animals in their care is an ongoing subject of debate: some place emphasis on the animals’ physical health; others, on animals’ ability to live “natural lives”; and others on animals’ affective states or psychological wellbeing. Recently, there has been an increase in interest in how an animal’s ability to exercise control over its environment could impact their welfare. In this article, we take the stance that the relevant aspects of the first two concepts of animal welfare (physical health, natural lives) are largely addressed when an animal’s psychological wellbeing is prioritized. Through that lens, we review the current state of the literature regarding the psychology of control, and the intersection between choice, control, and welfare. We clarify terms to support future work, and propose future directions that could lead to a better understanding of the psychological benefits of choice and control and ultimately inform animal care decisions.

## 1. The multidimensional concept of animal welfare

The concept of animal welfare is a complicated one. There is some philosophical controversy about how welfare should be defined, and care for an animal can differ dramatically depending upon which definition of welfare one adopts (1).

Veterinarians and farmers have historically placed emphasis on animals’ physical health in conceptions of welfare [e.g., (2–4)]. In other words, animals that were free from disease, injury, and illness, and were able to effectively produce and reproduce were considered to have good welfare. A predominant criticism of this view is that animals could be in excellent physical form but suffer psychologically (5–7). Others have proposed that the best means by which to promote good welfare is to enable animals to live “natural” lives, that is, by allowing animals to engage in the full range of behaviors that are expressed in their non-managed (wild) environment [e.g., (8–10)]. But it has been since been argued that definitions that rely on the expression of natural behaviors are also insufficient [see (11), or (12) for reviews], because some natural behaviors would be distressful and incongruent with our common sense of good welfare (e.g., a prey animal being chased by a predator), and some *unnatural* behaviors are rewarding to animals and support good health and positive experiences [e.g., engaging with computer tasks; (13)].

A third perspective, and perhaps the dominant perspective now, focuses on affective states, or “feelings”, of animals as central for determining animal welfare [e.g., (1, 5–7, 11, 14)]. Scientists in the “feelings” camp argue that the relevant aspects of the other two definitions (physical health, expressing natural behaviors) would often automatically be addressed if animals’ psychological needs are met, making those definitions are redundant. In other words, an animal that is wounded or ill would likely experience negative affective states, and therefore experience poor welfare (7). Similarly, others argue that so-called natural behaviors have little connection to welfare in and of themselves; any welfare impact stems from the effectiveness of behaviors (natural or otherwise) in promoting positive mental states (11, 15). Arguably the most prominent current model of animal welfare is the Five Domains Model, which considers health and behaviors relevant to an animal’s welfare state are dependent upon how the mental domain interprets or processes them (16, 17). We share the view that animals’ psychological states are the most important factor for determining welfare, and we propose that providing captive animals with opportunities to exercise control (i.e., via choices) is an excellent mechanism by which to improve psychological wellbeing and enhance welfare.

## 2. The intersection between choice, control, and welfare

Dawkins (18, 19) proposed that the simplest way to ensure that animals experience good welfare is to keep them healthy and give them what they want. Providing animals with choices seems a straightforward way to satisfy these requirements. First, providing choices can directly positively impact animals’ affective states by giving animals a sense of control and agency, which is imperative for psychological and biological health [see (20) for a review]. Second, theoretically, providing choices minimizes the role of human caregivers as deciders of what it is that animals want, instead allowing animals themselves to seek out their preferred alternatives (13, 19, 21). But before we can discuss these ideas further, we must first consider what is meant by choice and control.

### 2.1. The difference between choice, control, and related constructs

**Figure 1:**
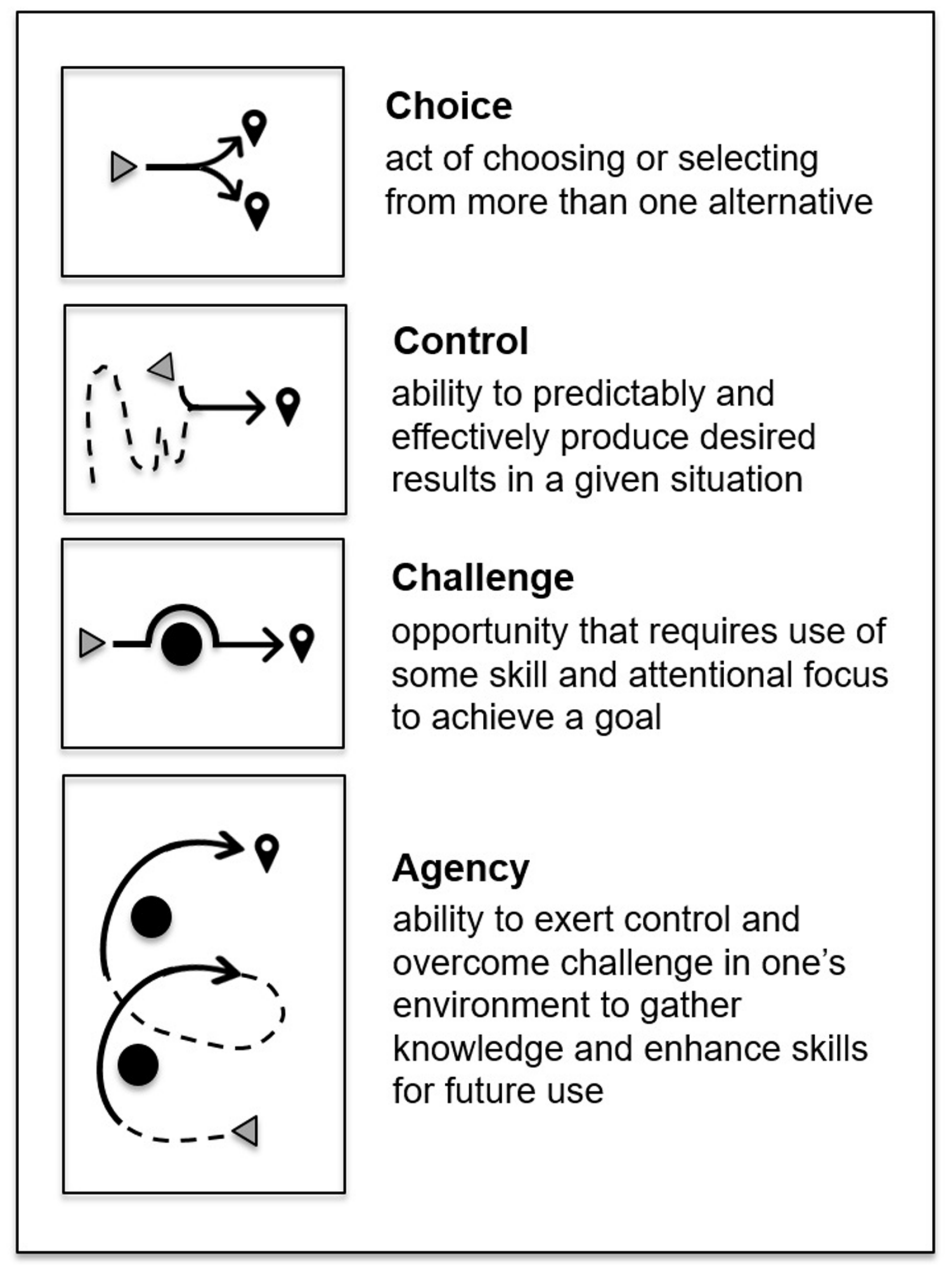
Conceptual definitions of choice, control, challenge, and agency. The organism/individual is represented by gray triangles, potential actions of the organism are represented by black arrows, and potential outcomes are represented by black location markers. The dashed lines represent (unpredictable) environmental factors, and the solid black circles represent obstacles (physical or mental).

To set the stage for empirical investigation of the relationship between choice and welfare, there is a need to disentangle some related constructs (Figure 1). We define *choice* as the act of choosing or selecting from more than one alternative (22). *Control* is the ability to predictably produce desired results – i.e., to be the cause that instantiates an expected effect (23) – and is related to the concept of autonomy (24, 25). It follows that choice is a main mechanism by which individuals can exert control over their environment (20). A similar, related construct is that of *challenge*. A challenge is an opportunity that requires the use of some skill and focused attention in order to achieve a goal, and has been argued to promote an animal’s sense of competence (26–28). Autonomy (i.e., the ability to exercise *control*) and competence (i.e., the ability to overcome *challenge*) are considered innate psychological needs for humans (24, 25), and from an evolutionary perspective, it follows that these needs should also be satisfied for positive psychological wellbeing in nonhuman animals who, like humans, evolved to be functional agents in unpredictable environments (20, 27).

Specifically, we propose that control and (surmountable) challenge are necessary for an animal to experience *agency*. Agency has been defined as the ability to successfully engage with the environment beyond satisfying immediate needs – that is, by achieving goals, developing skills, acquiring information, and pursuing future plans (26, 27). Agency is relevant to welfare because engaging in such intrinsically-motivated behavior is inherently rewarding (24, 28). Furthermore, building competency through agency allows for better coping with later difficult situations (26), and the ability to cope with the environment is directly related to an animal’s welfare state (4). But individuals only reap the benefits of competence if it is accompanied by a perceived “internal locus of causality” (i.e., control) over the situation at hand (24, 25, 29). Animals would likely have little motivation to engage in any sort of challenge if they had no control over the environment around them (20).

In interim summary, to promote positive welfare in animals, we must strive to increase the perception of agency. Špinka (26) and Špinka and Wemelsfelder (27) have provided excellent explanations for why challenge is integral to agency and necessary for good welfare. We argue that another critical component of agency is the ability to exert control over one’s environment through choices. Many studies, described below, have demonstrated the psychological relevance of environmental control, although fewer have investigated the direct welfare implications of providing *choice* to animals.

### 2.2. Control as a psychological imperative

Leotti et al. (20) argued that perceiving control over one’s environment is evolutionarily adaptive because increased control over the environment will improve an animal’s chance of survival. When faced with choices, animals are likely to choose options that provide them with the most benefit and avoid options that may cause harm. Leotti et al. (20) proposed that making choices is the main mechanism by which animals can exercise such control, and that having access to choice has evolved to become a biological imperative. The authors reviewed evidence that control is critical for typical, healthy development, and that a diminished sense of control can lead to maladaptive behaviors and reduced wellbeing (20).

Although Leotti et al. (20) discuss the importance of choice in enabling animals to experience control, the majority of research on control has investigated not choice behavior, *per se*, but the ability to start or stop aversive or rewarding stimuli. For example, Seligman and Maier (30) demonstrated that dogs who were repeatedly exposed to inescapable shock later did not attempt to escape electric shock even when it was avoidable, a phenomenon dubbed as “learned helplessness”, which has since been evidenced in a wide variety of species, from flies (31) to humans (32). Additionally, Hanson et al. (33) found that monkeys who could press a lever to avoid an unpleasant noise exhibited similar plasma cortisol levels as monkeys who were never exposed to the noise, whereas monkeys who did not have control over the noise exhibited elevated cortisol levels. Research on the positive effects of controllability over rewarding stimuli have also been found: Buchanan-Smith and Badihi (23) demonstrated that marmosets experienced improved welfare (as evidenced by positive activity patterns and scent marking) in the presence of supplementary light and/or heat, and that these effects were further enhanced when marmosets were given control over the light/heat source.

Research that has investigated control via choice, rather than stimulation management, has demonstrated that many species of animals prefer having two options available rather than one, even where there was no difference in reward [e.g., pigeons, (34); rats, (35); and monkeys, (36)], and that providing choices improves motivation and performance. For example, Washburn et al. (37) found that rhesus monkeys performed better on computer tasks when they were able to choose the tasks compared their performance on those same tasks when they were assigned. Beran et al. (38) found that capuchin monkeys demonstrated improved task performance when the monkeys only chose the *order* in which they completed tasks (not the tasks themselves) and even when the assigned order was yoked to match animals’ established preferences. Interestingly, the monkeys who demonstrated improved performance in the Beran et al. (38) study did so even though they had access to enriched living arrangements which already provided them with ample opportunities for choice and control.

So far, we have discussed the literature that makes clear that control is essential for positive psychological welfare, and that making choices is a means by which to exercise control. Less is known about the direct link between the provision of choice and psychological wellbeing in animals, though the research is promising. Badihi (39) found that increase in complexity, choice, and control resulted in decreases in undesirable behaviors (e.g., agitated locomotion, and scratching) and increases desirable behaviors (e.g., calm locomotion, exploration, and play) for marmosets. Kurtycz et al. (40) demonstrated that gorillas and chimps exhibited evidence of more positive affective states when given a choice between indoor and outdoor habitats compared to when no choice was given. Similarly, Ross (41) found that polar bears demonstrated decreased stereotypic behaviors and increased social play when they were provided access to private den areas compared to when no such option was provided. The polar bears used the dens only 2% more of the time when they were accessible, suggesting that the welfare benefits were a byproduct of the provision of choice rather than the dens themselves. And, research with humans has shown that anticipating the opportunity to make a choice engages reward-related circuitry in the brain (42).

There is also indirect evidence from choice studies that suggests the provision of choice has potential welfare benefits. Specifically, several studies have demonstrated that making choices serves as a primary reinforcer, such that animals enjoy making choices for the sake of making choices. Perdue et al. (43) found that, given the choice between two icons, one which allowed the monkeys to choose the order in which to complete tasks, and another which would force them to complete the tasks according the order assigned by the computer, monkeys preferred to choose the order in which to complete the tasks. Monkeys maintained this preference for choice even when the computer-assigned order was yoked to match their previous choice selections. In other words, the monkeys preferred making choices even when making such a choice did not lead to greater reward or more preferred outcomes than the non-choice scenario. Perdue and Brown (44) also found that monkeys chose less-preferred alternatives simply to keep those options available. Monkeys could choose between two icons that provided different levels of risk but the same overall rate of reward. After each choice, the unselected icon shrank in size until disappearing entirely (whereas choosing the icon returned it to full size). Perdue and Brown (44) found that capuchin monkeys would periodically choose their *less-preferred* icon to keep it from disappearing entirely. Together, this research speaks to the intrinsic value of having access to choices that goes beyond the value of the options themselves.

Finally, as Dawkins noted, asking animals what they want is key to supporting good welfare (18, 19). Even if choices were not rewarding in their own right, giving animals options would allow them to choose preferred alternatives, and tell us what things are most necessary or beneficial for their own wellbeing. After all, preferences can change over time (45) and options may still be valuable to animals even if they are not actively being used (21, 40, 41). This idea was nicely summed up by Washburn (13): “Rather than making assumptions about the conditions that will promote psychological wellbeing, we should endeavor to give the animals choices that will indicate those conditions… Allowing animals to indicate their preferences by their behavior provides the clearest and most defensible standard for determining environmental enrichment”.

## 3. Future directions

With concepts clearly defined and an understanding of what we have learned so far, some high priority research questions emerge. Making progress on the questions below will help lay the foundation for incorporating choice into animal care in a way that promotes good animal welfare.

### 3.1. What is the most beneficial frequency, abundance, and duration of choices?

Although the literature clearly demonstrates that at least some choice is more desirable and leads to better psychological outcomes than no choice, the relative influence of increasing the number or duration of such choice is less clear [though see (39)]. The law of diminishing marginal utility (46) would suggest that each additional alternative would be less valuable than the last, and research with humans has indicated that too many choices can lead to suboptimal outcomes (47, 48). It is unclear whether other animals may be susceptible to choice overload (49) or would experience a similar diminished marginal utility for a greater number of alternatives. A fruitful avenue of future research may be to investigate whether there may some optimal number of alternatives (at least in some contexts or for some species), where the perception of control is maximized but the increased number of alternatives does not become trivial or overwhelming. Furthermore, it will be important to investigate the possible consequences of removing choice for a duration of time after it has been established, as this is a practical likelihood for animals in human care.

### 3.2. How should natural history inform what and how choices are provided?

The majority of research on choice and control so far has been conducted with primates. Although we hypothesize (and evidence supports) that all animals would reap benefits from being functional agents in their environment, the psychological impact of choice may differ from species to species. Most primates evolved to live in groups with complex social dynamics, consume varied diets, and inhabit heterogenous environments. These traits could make primates especially sensitive to the availability of choice in their environment; or, it is possible that we are better prepared to consider the value of choice for primates due to a bias in our science and publication history (50, 51). Certainly, though, natural history must be considered in *how* and *what* choices are provided, as well as in how “good welfare” would be measured (52). More research on choice behavior in non-primate species, particularly understudied species such as reptiles, amphibians, fish, or invertebrates, is needed, as well as strategies for interpreting their responses (19).

### 3.3. How do we measure the benefit of unselected choices?

Generally, the value of alternatives has been measured based on the *active use* of the chosen alternative. Only recently [e.g., (21)] have scientists begun asking what the value could be of alternatives that are not actively being used. Research suggests that choices are valuable even when they aren’t chosen or aren’t preferred [e.g., (40, 41, 43)]. However, there are practical constraints to the number of choice opportunities that can be provided to animals in human care. Using work-for-access paradigms could shed some light on the relative value of differing alternatives. For example, a study on American minks showed that the animals spent 300% more time in nest boxes than swimming baths when both options were available (53), yet the animals worked much harder for access to their swimming bath than the nest box, and only showed increased cortisol levels when being denied access to the bath, not the nest box (54). Incorporating more research that requires animals to work for access to certain options (or to having the opportunity to make a choice at all) could help us answer questions about when choice is most important, which options are most valuable to individual animals, how those values change over time or in different contexts.

## 4. Conclusion

In this article, we have reviewed the literature on welfare, choice, and control, and discussed the ways in which these topics intersect. The majority of research has investigated the role of control on psychological wellbeing via stimulation management. However, we also know that making choices is a means by which to exercise control, and that studies on choice behavior have indicated that making choices can have a direct positive impact on welfare. Yet gaps in the literature remain, including how non-primates are affected by choice opportunities, the impact of the duration of choice or number of options available, and the benefit of unselected alternatives. We are optimistic that providing choices to captive animals will continue to prove an excellent strategy to improve their welfare while also enabling us to learn about their preferences, choice behavior, and cognition more generally.

## Author contributions

ME and KC developed the concept of the manuscript and reviewed and revised the article. ME wrote the first draft. All authors contributed to the article and approved the submitted version.

## Funding

This work was supported by Lincoln Park Zoo.

## Conflict of interest

The authors declare that the research was conducted in the absence of any commercial or financial relationships that could be construed as a potential conflict of interest.

## Publisher’s note

All claims expressed in this article are solely those of the authors and do not necessarily represent those of their affiliated organizations, or those of the publisher, the editors and the reviewers. Any product that may be evaluated in this article, or claim that may be made by its manufacturer, is not guaranteed or endorsed by the publisher.
